# Insights from the salt bridge analysis of malate dehydrogenase from H. salinarum and E.coli

**DOI:** 10.6026/97320630015095

**Published:** 2019-02-28

**Authors:** Amal Kumar Bandyopadhyay, Rifat Nawaz Ul Islam, Debanjan Mitra, Sahini Banerjee, Saba Yasmeen, Arunava Goswami

**Affiliations:** 1 Department of Biotechnology,The University of Burdwan,Burdwan, West Bengal,India; 2 Department of Zoology,The University of Burdwan,Burdwan,West Bengal,India; 3 Department of Biological Sciences,ISI,Kolkata,West Bengal,India; 4 Department of Botany and Microbiology,Acharya Nagarjun University,Nagarjun Nagar,Andra Pradesh,India

**Keywords:** Malate dehydrogenase, Stability, Halophilic, Salt Bridge, Sequence, Homology model

## Abstract

Halophilic proteins have greater abundance of acidic over basic residues in sequence. In structure, the surface is decorated by negative charges, with lower content of Lysine. Using sequence BLOCKs and 3D model of malate dehydrogenase from halophilic archaea (Halobacterium salinarum; hsMDH) and X-ray structure from mesophilic bacteria (E. coli; ecMDH), we show that not only acidic and basic residues have higher mean relative abundance (MRA) and thus, impart higher polarity to the sequences, but also show their presence in the surface of the structure of hsMDH relative to its mesophilic counterpart. These observations may indicate that both the acidic and the basic residues have a concerted role in the stability of hsMDH. Analysis on salt bridges from hsMDH and ecMDH show that in the former, salt bridges are highly intricate, newly engineered and global in nature. Although, these salt bridges are abundant in hsMDH, in the active site the design remains unperturbed. In high salt where hydrophobic force is weak, these salt bridges seem to play a major role in the haloadaptation of the tertiary structure of hsMDH. This is the first report of such an observation.

## Background

Apart from normal mesophilic environment, microbes also exist in the extreme of physical and chemical conditions. Halobacteria are archaea that thrive under highly saline brine conditions [1,2]. These microbes grew up with specialized transport-devices in their cell membrane to maintain isomolarity of saturated salinity of the cell as environment. In the cytoplasm of cell, K+ concentration is 4-5 times than that of Na+ [3]. As a consequence, soluble proteins and enzymes are maintaining functionality and stability under this condition, which is known to be deleterious for mesophilic counterparts [3,4]. Comparative analysis of halophilic and mesophilic proteins showed abundance of acidic over basic and lower bulky hydrophobic residues in the sequence of the former [3, [5,6]. Experiments on ferredoxin and hsMDH from Halobacterium salinarum showed that withdrawal of salt from the medium induce unfolding of the protein [2,7,8], which can be partially resisted by lowering the pH of the medium [7]. At intermediate salt (~0.5M to 1M NaCl), ferredoxin forms molten globule like state, which differs in stability from either the native state (~4.3M NaCl) or low salt form (~0.05M NaCl) [9]. Similar structural instability and loss of enzymatic activity are observed for many halophilic proteins and enzymes [5,7,10]. Except few cases [11], analyses of atomic structures from halobacteria showed cluster of negatively charged residues in the surface [10,12,13,14,15]. Binding of solvated ions has also been another halophilic strategy for multimeric halophilic enzymes [12,16]. Halophilic protein has reduced hydrophobic surface, which is not due to hydrophobic but due to Lysine residue [17]. As far as salt bridge is concerned, in malate dehydrogenase (hmMDH) from Haloarcula Marismortui [1], inter-dimer and intermonomer ion-pairs has been observed that resists subunit dissociation [1]. Using crystal structure database, it has been shown that on average the content of stable intra-subunit or monomeric salt bridges are double in halobacteria than that of mesophilic microbes [18,19].

hmMDH (UNIPROT ID: Q07841) is a soluble protein that takes part in reversible dehydrogenation of malate into oxaloacetate with concomitant formation of reducing equivalent (NADH). The enzyme is extensively studied using varieties of in vitro experiments [1,10,20] to address the details of salt dependent properties. The sequence of hmMDH has 27% identity with the acidic and the basic residues are double (20.4%) and equal (9.9%) to that of E coli malate dehydrogenase (ecMDH, UniProt ID P61889) respectively. Similarly, malate dehydrogenase from Haloferax volcani (hvMDH; UniProt ID: Q9P9L2) has 27.1% identity with ecMDH and acidic and basic residues constitute 19.1% and 9.5% respectively. Using biochemical and crystallographic studies, it has been shown that excess acidic residues interact with water molecules and salt ions in the surface of the protein and that contribute to the haloadaptation of the protein [20,21].

hmMDH reveals a] highly acidic surface, b] excessive interactions of the protein with solvated ions and water molecules and c] excessive inter subunit salt bridges. How does a subunit of the protein (i.e. tertiary structure) remain stable in these unusually high salt conditions? The fact that solvent property is severely affected in high salt conditions, the balance of weak interactions in halophilic proteins remains to be understood relative to its mesophilic homologues. The fact that non-specific electrostatic interactions have less contribution to the overall stability of protein [5], excess charges seem to have additional role in the overall stability of the protein. Reduction of net hydrophobic force would be a consequence in high salt due to low water activity situation [22].

Here, we report results of sequence and structural studies on monomeric malate dehydrogenase from Halobacterium salinarum (hsMDH) in comparison to its mesophilic homologue (ecMDH). The study highlights the details of alteration of mean property of sequences of hsMDH and its implication in the stability and functionality of the protein. Further, the role of specific electrostatic (salt bridges) in relation to evolutionarily acquire acidic and basic residues has also been investigated in this work. We then discuss our results with state-of-the-art understanding of the field. Overall, our analyses reveal new insight in the adaptation of hsMDH, which we believe would have potential applications in structural bioinformatics.

## Methodology

### Dataset

We performed detailed studies on sequence and structure of hsMDH in comparison to mesophilic homologue. For sequence study, we extracted sequences of halophilic archaea and mesophilic bacteria in FASTA format from UniProt database for comparative analysis. As the 3D structure of hsMDH is not available, we procured structure of Haloferax volcanii, hvMDH (4BGU) from the Research Collaboratory for Structural Bioinformatics (RCSB) protein data bank (PDB) [23], which was used as template for the development of homology model structure of hsMDH.

### Construction and evaluation of model

To date there are 10 structures of malate dehydrogenase from Haloacrula marismortui (hmMDH; 9) and Haloferax volcanii (hvMDH; 1) in RCSB, PDB database. The model of hsMDH (UniProt ID: Q9HMV8) is developed against the template structure of hvMDH (PDB ID: 4BGU; UniProt ID: Q9P9L2) using Modeller 9v11 in-built scripts as earlier [24, 25, 26, 27, 28, 29]. Use of the latter structure as template over the structures of hmMDH is justified as hvMDH has 83% sequence identity (which is 6% higher than hmMDH) with hsMDH and that the crystal structure of hvMDH, 4BGU has highest resolution (1.5 Å) of any known crystal structure of halophilic malate dehydrogenases in the database. Alignment between the template (4BGU_A, 303 amino acids) and the target (Q9HMV8, 304 amino acids) was performed as earlier [24, 25,26] along with manual improvements [24, 25, 27, 30]. Final model was selected based on the Discrete Optimized Protein Energy (DOPE) score. The model was refined using AUTOMINv1.0 [31]. Final model was evaluated as earlier [24, 26, 27, 28, 29] along with ion-pairs [25].

### Physicochemical and sequence properties

Separate FASTA files (with sequences >100) of malate dehydrogenase from halophile and mesophile were subjected for the preparation of BLOCK using ABPT tool of PHYSICO2 [32] and the BLOCKs were then analyzed using web tools [32,33]. Mean relative abundance was computed from the computed mean value for a given physicochemical property using the following formula.

 MRA (Mean Relative Abundance) = (Mean value of hmMDH - Mean value for ecMDH)/ Mean value for ecMDH 

Mean Kyte-Doolittle hydrophobicity was computed from the positional values of candidate sequences and plotted against sequence position. Shannon entropy (for hmMDH only) was computed and plotted to check positional variability. All these results were readily obtained by the use of PHYSICO2 [32].

### Salt bridge extraction and analysis

Analysis of salt bridge was performed on minimized structures of hmMDH and ecMDH using web-program [34,35]. To check the overall connections, partners of isolated and networked salt bridges are linked on aligned sequences of hmMDH and ecMDH.

## Results

### Structural features of the model of hsMDH

At low salt conditions, multimeric halophilic proteins undergo subunit dissociation [3]. In these aspects, hmMDH has been investigated in details [1, 10,20]. PDB database contains nine structures of hmMDH that are either in dimeric or tetrameric forms. How does a monomer (i.e. the tertiary structure) maintain stability in high salt conditions? Weak interactions form the tertiary structure. Hydrophobic force would be weak under halophilic conditions due to low activity of water [3]. Is there an alternate strategy for the stabilization of the tertiary structure? We, therefore, developed 3D model structure of hsMDH to perform comparative analysis.

**Figure 1:**
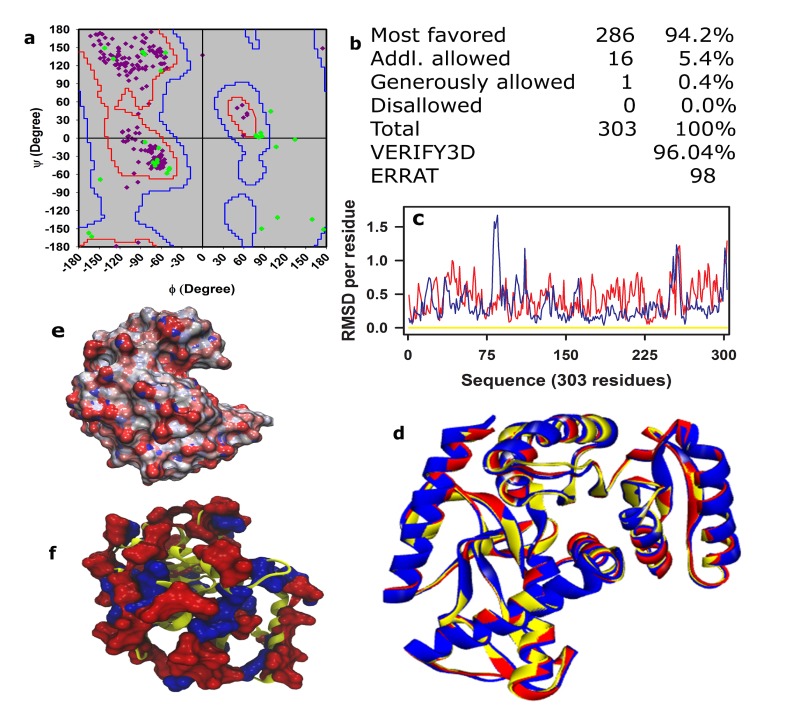
Evaluations and surface characteristics of the model of hsMDH. Evaluations are performed using (a) Ramachandran plot, (b) quantitative distribution values of Ψ and Φ for different regions using PROCHECK [36], (c) per residue RMSD and (d) average RMSD. Average and per residue RMSD that compares model structure (red; d) and 4CJO (Blue, hmMDH; d) with reference structure 4BGU (template, yellow; d) using VMD [37]. Electrostatic surface potential of the model structure, which was obtained using APBS [38] at neutral pH, was projected onto the molecular surface of the model (e). The distribution of negative (red) and positive (blue) charged residues of the model are shown in (f).

The details of development of the model of hsMDH against the template (4BGU), minimization and evaluation are performed as earlier [24, 25, 26, 27, 28, 29]. The main chain dihedral angles (Φ and Ψ) are seen to occupy the favored region of the Ramachandran plot (Figure 1a,b). Almost equivalent score for non-bonded interactions and 3D-1D (at ≥ 0.2 levels) (Figure 1b) are obtained for the model (of hsMDH) and the template (Figure 1b). The root mean square deviation (RMSD) measures the distance in angstrom between the Cα-atoms of superimposed proteins. To check the main chain topology of the model, we have compared average and per residue RMSD of the model and wild-type hmMDH i.e. 4JCO (Figure 1c, d) using template as reference. The superimposed structures (Figure 1d) of the model (red) and 4JCO (blue) show very low average RMSD i.e. 0.539 and 0.440 respectively in reference to the template (Figure 1; yellow trace). Conformational fluctuation of the model structure (Figure 1; red trace) and that for the 4JCO (Figure 1; blue trace) are judged at residue level against the template structure (Figure 1c) using per residue RMSD. Overall, per residue RMSD of the model is seen to be lower than 1Å for the entire residues range of the protein (303 residues). Further, like hmMDH, the surface of hsMDH is seen to be decorated with negative potentials and charges (Figure 1e, f). Further and remarkably, the salt bridge interaction pattern of the model and the template remain almost equivalent (>95%) to each other (data not shown). Taken together, it could be said that the present model structure is well formed as it passes all the above evaluation criteria.

**Figure 2:**
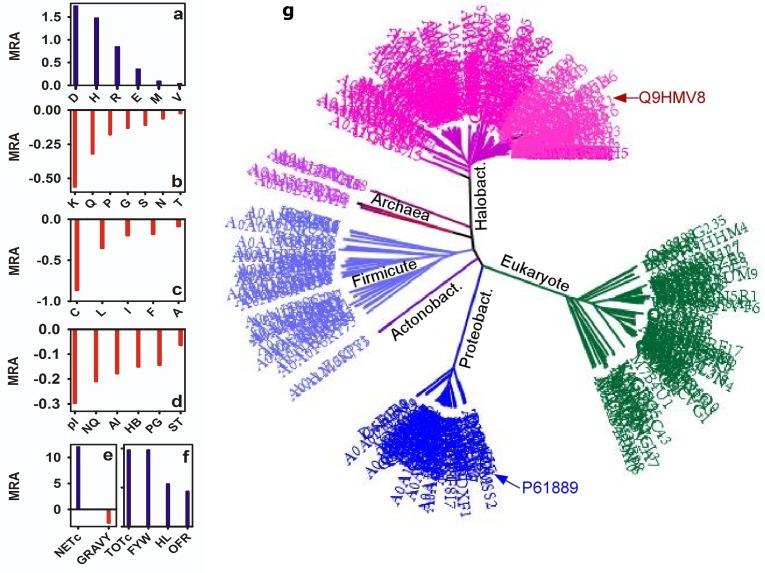
Mean relative abundance (MRA) for residues (a-c) and some of its classes (d-f) along with the phylogenetic tree (g). In the latter, different clades are identified by colors of which P61889 and Q9HMV8 denote ecMDH and hsMDH respectively

ecMDH (P61889) and hsMDH (Q9HMV8) are distantly related Unlike E. coli (mesophilic, ecMDH) malate dehydrogenase (3HHP), halophilic enzyme functions in saturated salt solution [1]. Analysis of difference matrix showed that hsMDH has only 27.6% identity with ecMDH (Figure 2g). The MRA of D, E (acidic) and H, R (basic) are higher in hsMDH (Figure 1a). Total charge (TOTc) and net charge (NETc) are also higher (Figure 1e and f) in hsMDH. Interestingly, the MRA of polar (S, T, N, Q, P, G) and LYS (K) residues are lower in hsMDH. Although MRA of polar residues are lower, hydrophilic (HL) and aromatic (F, W, Y) residues are higher in the protein (Figure 1f). Due to the latter, order forming residues (OFR) is also higher in hsMDH. As far as hydrophobic residues are concerned, except M, V, which has slight positive MRA, others (C, L, I, F, A) are lower in hsMDH. Thus, mean GRAVY is lower in hsMDH. APBEST analysis [39] of BLOCK FASTA (n≥100) showed that the NCS:CS (non-conservative to conservative substitution ratio) of hsMDH is 0.38 and that in the ecMDH is 0.51.

### Distribution of SB forming residues in the surface and core of hsMDH

Why are acidic (D, E) and selective basic (H, R) but not polar residues show higher MRA in hsMDH? What are the relative distribution of these residues in the surface and core of the protein? To check this, we have presented absolute and normalized distribution of acidic (D, E) and basic (H, R, K) residues, that are i] present in the protein (P) and ii] participating in the salt bridge (SB) formation, of hsMDH and ecMDh in Table 1. The details of normalization are mentioned in the table. For example, in case of ASP of ecMDH, out of 12 ASP (100%), SB total is 4 i.e. (4*100)/12=33.3%. SB su = (33.3*2)/4 = 16.7% and SB co = (33.3*2)/4 = 16.7%. This would mean, 33.3% of ASP is present in the SB of which 16.6% each in the core and surface. Similarly, normalization of the surface and core distributions of SB is computed for other residues. Several points are noteworthy from the table.

First, in protein (P) and in SB, higher abundance of ASP and GLU are observed in the surface of hsMDH. Second, HIS has similar type of distribution preference in the surface of hsMDH. Notably, HIS is much higher in hsMDH. Third, ARG has distribution preference in the core of hsMDH in reference to ecMDH. Forth, LYS shows preference for both the surface and the core in the case of SB residues but not with respect to the protein (Table 1). Fifth, although LYS is far less in hsMDH, both ARG and LYS are fully (100%) utilized for the formation of salt bridges. In an earlier study, surface reduction of LYS was observed in glucose dehydrogenase [17], which has been the halophilic strategy for the reduction of hydrophobic characteristics of the surface in the protein. Here we see, although LYS is far less in hsMDH, 100% of it is used for SB formation, which got preference both in the surface and in the core of hsMDH (Table 1). Finally, although acidic residues are seen to preferentially decorate the surface of hsMDH as has been observed in other halophilic proteins [1, 20, 12, 13, 14, 15], basic residues also show similar preference in the surface and in the core of hsMDH.

### Salt bridge partners alter sequence properties

**Figure 3:**
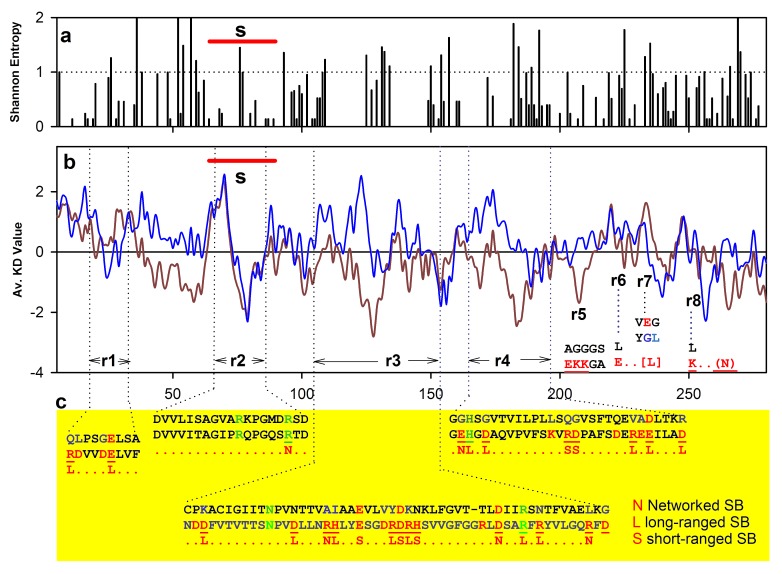
Shannon entropy (a) of hsMDH, mean Kyte-Doolittle hydrophobicity (b) properties (red hsMDH and blue ecMDH) and region (c) r1, r2 etc of sequence specific salt bridges in hsMDH relative to ecMDH. s indicates substrate specificity site.

The GRAVY of the hsMDH and ecMDH are -0.2 and 0.2 respectively. Surprisingly, MRA of polar residues (N, Q, S, T, P, G) of hsMDH is lower than that of ecMDH (Figure 2b) indicated hydrophilicity in hsMDH is largely contributed by acidic and basic residues. Except LYS, MRAs of D, E and H, R are higher in hsMDH (Figure 2b). How does the charged residues mediated polarity affect sequence property? To check this, we have computed Kyte- Doolittle mean hydrophobicity for malate dehydrogenases and plotted in Figure 3. Several points are noteworthy from the figure. First, except the substrate specificity site (Figure 3; s), other sites (r1-r8) are more hydrophilic in hsMDH than ecHMD, which is largely due to acidic and basic residues. Increase of these residues is resulted from homologous substitutions. Second, most of these substituted acidic and basic residues are seen to form salt bridges of isolated and networked (short and long ranged) types (see below). Substituted polar residues are not as global as these charged residues. Finally, the sequence property of hsMDH is seen to be largely determined by acidic and basic partners of salt bridges.

### Binary properties of salt bridge in hsMDH relative to ecMDH

**Figure 4:**
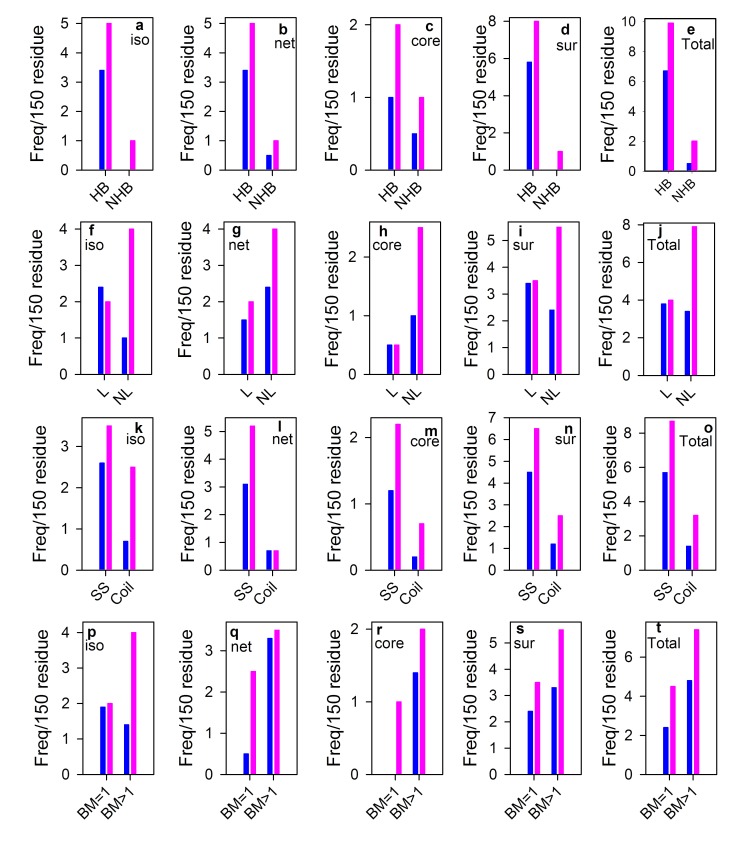
Binary properties of salt bridge of hsMDH (pink) relative to ecMDH (blue). Binary items of isolated (iso; a, f, k, p) vs networked (net; b, g, l, q) and core (c, h, m, r) vs surface (sur; d, i, n, s) are separately plotted along with total frequency (e, j, o, t). As the residues are unequal in these two proteins, normalization was done using earlier scale [40]. HB hydrogen bonded; nHB non-hydrogen bonded; L local; nL non-local; SS salt bridge in secondary structure (Helix/strand); BM bond multiplicity

It is seen in the figure (Figure 4) that almost all binary items are increased in hsMDH relative to ecMDH. Not only isolated but also networked salt bridges increase both in the core and surface of hsMDH. Remarkably, non-local, hydrogen bonded, secondary structured and multiple bonded salt bridges are far greater in hsMDH than that of ecMDH (Figure 4). In hsMDH, newly designed salt bridges are also seen (Figure 4a, d and r), which are completely absent in ecMDH.

### Salt bridge in hsMDH is more circuitous than ecMDH

**Figure 5:**
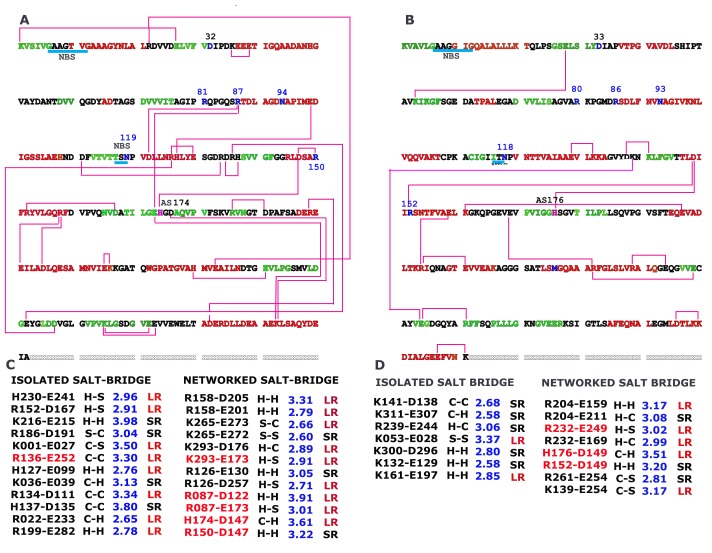
Interconnection of acidic and basic partners of salt bridge on aligned sequence of hsMDH (A) and ecMDH (B) along with details of salt bridges (C for hsMDH and D for ecMDH). Buried type salt bridges are indicated by red color (C and D). Sequence color indicates secondary and coiled structures in that red indicates helix, green indicates strand and black indicates coil. SR short-ranged; LR long ranged; H helix; S strand; C coil. NAD binding regions are identified by cyan underline and NSB. Blue numbers (81, 87, 119 and 150) indicate substrate binding residues. Residues 32 and 94 indicate NAD+ binding residues. AS represent active site that takes part as proton acceptor

In hsMDH, partners of salt bridge make more intricate connections (Figure 5A) than that in ecMDH (Figure 5B). In the above, we have shown that these salt bridge forming residues play major role in altering the sequence property of hsMDH (Figure 3b). Compare to ecMDH, hsMDH forms 24 salt bridge pairs of which 70% are long ranged with 12 pairs are networked type. Further, buried salt bridges are also increased in hsMDH (Figure 5C). NAD(+), substrate binding sites and active site (that takes part in the proton exchange mechanism) are seen to be involved in the formation of additional salt bridges in hsMDH, which are absent in ecMDH (see below).

### Newly designed salt bridges in hsMDH

**Figure 6:**
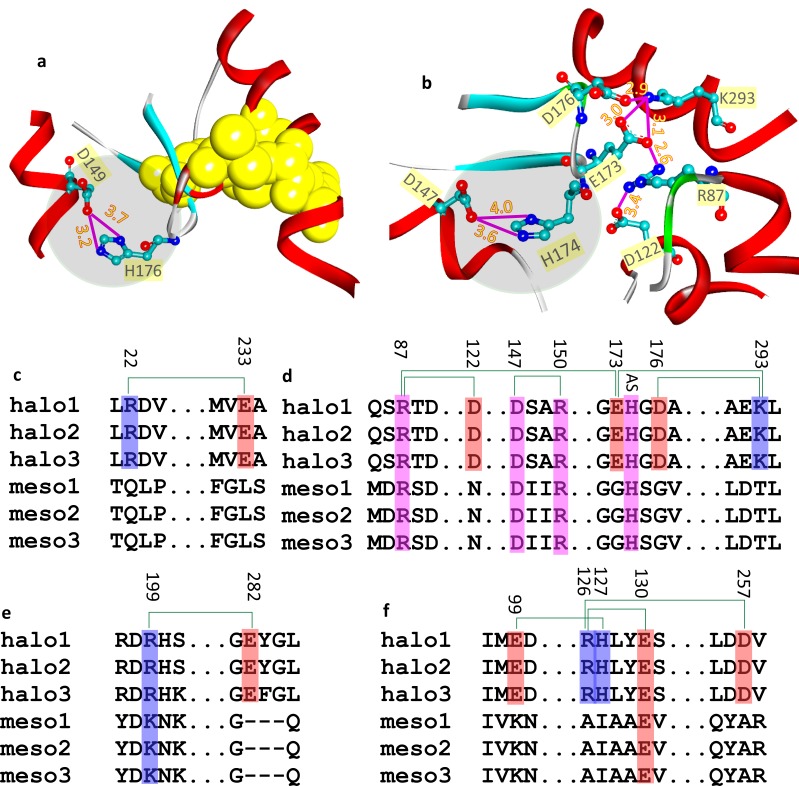
Salt bridges of ecMDH (a) and hsMDH (b) at the proton acceptor site (round and grey shade region) along with stabilizing hydrophobic and SB interactions respectively at its proximity. The salt bridges near the active site of hsMDH are due to non-conservative substitution (c), conservative substitutions at the active site (d), acidic insertion (e) mixed type substitutions (f).

hsMDH forms buried and networked salt bridge in the vicinity of the active site (Figure 6b, d), which is constituted by substrate specificity site (R81, R87 and R150; Figure 5A), NAD(+) binding site (118-121) and proton acceptor site (H174). This site (i.e. site H174) is common for both ecMDH and hsMDH that forms identical salt bridge (Figure 6a and b). The residue positions at the active site are seen to be conserved. Segments associating these residues forming open complex structure for ecMDH and hsMDH (Figure 6a and b). β-hairpin loop that have proton acceptor residue (H174) in hsMDH also harbors E173 and D176 residues. K293 and D122 containing helices are close to the active site. These E173, D176, K293 and D122 are halophilically substituted residues that form buried and networked salt bridge, which is otherwise seem to be stabilized by nearby hydrophobic residues in ecMDH (Figure 6a, d). Moreover, unlike ecMDH, hsMDH also forms long-ranged salt bridges, which are resulted due to non-conservative (E233, Figure 6c and R126, H127 and D257, Figure 6f) and conservative (R22, Figure 6c and E99, Figure 6f) substitutions and insertion (E282, Figure 6e).

## Discussion

It appears that some hitherto unknown principle of evolutionary surges induced halophilic archaea to maintain a deliberate style of life in highly saline brine conditions, where a mesophilic organism is inaccessible. Over the course of evolution, it has installed specialized transport devices in its cell membrane that maintains isomolarity of saturated salinity inside and outside the cell [2]. Soluble proteins in the cytoplasm are functioning in this saturated salt solution [2], which is known to be harmful for many mesophilic proteins [15]. The interactions of hydrated salt-ions with the negative charges in the surface of hmMDH maintain the stability of protein that resists subunit dissociation in high salt [20]. Atomic structure reveals, relative to mesophilic homologue, higher level of inter subunit salt bridge interactions that maintain the stability of the quaternary structure of hmMDH [1, 10,20]. Since hsMDH is less studied and since salt dependent stability at the tertiary structural level is not yet known, we perform detailed investigation on sequence and structural aspects of the protein in comparison to its mesophilic homologue.

## Model of hsMDH is well formed

The model of hsMDH is constructed using hvMDH (4BGU) as template. The sequence identity of hsMDH and hvMDH (83%) is higher than hmMDH (77%). Further, the resolution of 4BGU (hvMDH) is also highest of any known structure of halophilic malate hedydrogenase in the RCSB PDB database. Model structure of hsMDH has qualified all the evaluation criteria. The main chain topology of the model is almost identical with that of the template as has been judged by RMSD (0.54 Å). Similarly, RMSD of the model and wild type hmMDH (4JCO) are also very low (0.44 Å). Investigation of loop characteristic of the substrate specificity site, proton acceptor site and NAD (+) bonding site indicated the model is typical of an open active site form. Notably, structure of ecMDH (3HHP) and hvMDH (4BGU) has the similar conformation of the active site as the model structure of hsMDH [41].

## Model of hsMDH is well formed

hsMDH is distantly related to its mesophilic homologue (ecMDH) To workout the halophilic preference of amino acids in the sequence of hsMDH, we computed mean relative abundance (MRA) of amino acid residues. Out of twenty amino acids, only seven residues have positive MRA, which includes D, E (acidic), R, H (basic), Y (aromatic, amphoteric) and M and V (hydrophobic) [42]. Positive MRA of both acidic and basic residues indicated that they might have some concerted role. Although, two of the hydrophobic residues have slight positive MRA, the GRAVY of hsMDH is negative and that for ecMDH is positive. Although, hsMDH is hydrophilic, none of the polar residues (N, Q, S, T, P, and G) has positive MRA, indicating that acidic and basic residues largely contribute the sequence polarity. It seems that the increase of polarity by acidic and basic residues is the secondary effect. The primary role of these residues seems to be related with the formation of ion-pair or salt bridge (see below). Further, comparison of homologous position shows 75% difference between hsMDH and ecMDH. Such difference is also reflected in the phylogenetic tree in that hsMDH and ecMDH belong to separate clades. The non-conservative to conservative substitution ratio (NCS:CS) is far lower in hsMDH than mesophilic MDH indicated that the divergence is more decisive in the former. Acidic and hydrophobic residues in halophilic and mesophilic MDH largely maintain CS respectively, suggesting that the functional constraints are differentially maintained in these clades.

## Salt bridge forming residues are abundant in both the surface and core of hsMDH

Halophilic adaptation shows some common structural features in that i] surface is decorated by clusters of acidic residues [1, 20, 12, 38, 10, 14, 15] and ii] reduction of hydrophobic patches in the surface of these proteins [17]. The latter is achieved by reduction of lysine residues but not by hydrophobic ones [17]. Our analysis on salt bridge forming residues of hsMDH and ecMDH suggest that not only acidic but also basic residues show higher relative abundance in the surface of the former. Further, basic residues (R, K) also show higher relative abundance in the core. Notably unlike ecMDH, basic residues in hsMDH are fully utilized in the formation of salt bridges. These observations may suggest that salt bridge plays crucial role in the stability of the tertiary structure of hsMDH.

## Salt bridge partners alter sequence properties of hsMDH and impart stability

Comparison of hydrophobicity profiles of hsMDH and ecMDH show discernable difference indicating the change of positional residues in these sequences. Closer observations on region specific substitutions reveal that majority of these are contributed by acidic and basic residues. Interestingly, these substituted acidic and basic residues are involved in the formation of non-local, isolated and networked salt bridges in the surface and core of hsMDH. It is worth noting here, the above mentioned evolutionary consequence of the substitutions has been the alteration of the sequence properties. As majority of these substituted residues are involved in the formation of salt bridges, we postulate that the direct effect of these acidic and basic substitutions is to renew the stability of hsMDH in high salt conditions. Notably, dielectric property of the solvent is lowered at saturated salt solution [43], which would affect the hydrophobic force. In turn, in high salt conditions, salt bridges are less affected [44]. The reduction of hydrophobicity and increase of salt bridges in hsMDH seems to be the evolutionary strategy for the maintenance of structure and stability of the protein in high salt.

## Salt bridges are globally engineered in hsMDH

Critical investigations on binary items of salt bridges show, in almost all cases, hsMDH has higher level than ecHDM. Secondary structures (helix and strand), which acts as determinant of the topology of protein, show higher content of salt bridges in hsMDH than ecMDH. Hydrogen bonded salt bridges that contribute more to the stability than non-hydrogen bonded ones, also show higher proportion in hsMDH. Again, non-local salt bridges, which are important for the maintenance of globular shape of proteins, are increased in hsMDH. Core and surface of hsMDH are decorated with additional isolated and networked salt bridges. Taken together, such higher levels of intricate salt bridges in hsMDH indicate that the detrimental effect of high salt [3] is largely compensated by the increased level of different types of salt bridge.

## Newly designed salt bridges in hsMDH without affecting the active site

The pattern of salt bridge in the active site of hsMDH and ecMDh are identical. At the same time, newly designed salt bridges are also introduced in the former, which might be due to the maintenance of conformation of the protein in high salt. Majority of the designed salt bridges are the result of conservative and non-conservative substitutions. Remarkably, in hsMDH, new salt bridge is also introduced by insertion of crucial acidic residue. Although, active site possesses identical design in hsMDH and ecMDH, buried and networked salt bridge has been incorporated at the substrate and NAD (+) binding site of the former. These salt bridges, which are the result of substituted acidic and basic residues, are absent in ecMDH but nearby bulky hydrophobic residues seems to be involved in the stability in this case. We therefore hypothesized that the new design of salt bridges seems to be an alternate strategy to hydrophobic force that imparts local (short-ranged salt bridges) and global (long-ranged salt bridges) stability, substrate specificity and functionality.

## Conclusion

Using sequence-BLOCK of malate dehydrogenase from mesophilic and halophilic domains of life, we demonstrate that acidic (D, E) and basic (R, H) but not the polar residues, have higher mean relative abundance in sequence. Using model structure of Halobacterium salinarum (hsMDH) and crystal structure of E. coli (3HHP), we show that the surface of the former not only has higher abundance of acidic but also has basic residues (H, K). R, K, which are fully utilized in the formation of salt bridge in hsMDH, show their abundance in the core of the protein. We infer that the primary effect of acidic and basic residues in hsMDH is the formation of salt bridge and the secondary effect is the change of sequence property in hsMDH. Although, salt bridges in hsMDH are newly designed to be highly intricate and global in nature, the active site design of salt bridge is maintained in the protein. Overall, these salt bridges in hsMDH seem to have direct relation with the adaptation of the protein in highly saline brine conditions.

## Conflict of Interest

none

## Figures and Tables

**Table 1:** Absolute and normalized distribution of acidic and basic residues of protein and that participates in the SB formation. hsMDH and ecMDH has 303 and 312 residues respectively. Thus, frequency of these residues of hsMDH is first scaled to, per 312 residues. Now for protein (P), the total absolute frequency is referenced to 100 and then, frequencies of protein surface (P su), protein core (P co) and SB total are computed. Normalization of SB su and SB co are done using absolute frequency of SB total as reference.

ASP	GLU	HIS	ARG	LYS
ecMDH	hsMDH	ecMDH	hsMDH	ecMDH	hsMDH	ecMDH	hsMDH	ecMDH	hsMDH
P total	12 (100)	35 (100)	20 (100)	24.7 (100)	2 (100)	6.2 (100)	8 (100)	13.4 (100)	21 (100)	7.2 (100)
P su	10 (83.3)	31.9 (91.2)	16 (80)	21.6 (87.5)	1 (50)	4.1 (66.7)	7 (87.5)	8.2 (61.5)	18 (85.7)	6.2 (85.7)
P co	2 (16.7)	3.1 (8.8)	4 (20)	3.1 (12.5)	1 (50)	2.1 (33.3)	1 (12.5)	5.1 (38.5)	3 (14.3)	1 (14.3)
SB total	4 (33.3)	11.3 (32.4)	11 (55)	13.4 (54.2)	1 (50)	4.1 (66.7)	7 (87.5)	13.4 (100)	7 (33.3)	7.2 (100)
SB su	2 (16.7)	8.2 (23.5)	7 (35)	10.3 (41.7)	0 (0)	2.1 (33.3)	6 (75)	8.2 (61.5)	7 (33.3)	6.2 (85.7)
SB co	2 (16.7 )	3.1 (8.8)	4 (20)	3.1 (12.5)	1 (50)	2.1 (33.3)	1 (12.5)	5.1 (38.5)	0 (0)	1 (14.3)
